# Characterization, Statistical Analysis and Method Selection in the Two-Clocks Synchronization Problem for Pairwise Interconnected Sensors

**DOI:** 10.3390/s20174808

**Published:** 2020-08-26

**Authors:** Juan-Antonio Fernández-Madrigal, Angeles Navarro, Rafael Asenjo, Ana Cruz-Martín

**Affiliations:** 1 Department of System Engineering and Automation, University of Málaga, 29016 Málaga, Spain; acm@uma.es; 2 Department of Computer Architecture, University of Málaga, 29016 Málaga, Spain; angeles@ac.uma.es (A.N.); asenjo@uma.es (R.A.)

**Keywords:** clock synchronization, networked sensor fusion and decisions, sensor applications

## Abstract

Time synchronization among sensor devices connected through non-deterministic media is a fundamental requirement for sensor fusion and other distributed tasks that need a common time reference. In many of the time synchronization methods existing in literature, the estimation of the relation between pairs of clocks is a core concept; moreover, in applications that do not have general connectivity among its devices but a simple pairwise topology, such as embedded systems, mobile robots or home automation, two-clock synchronization is actually the basic form of the time estimation problem. In these kinds of applications, especially for critical ones, not only the quality of the estimation of the relation between two clocks is important, but also the bounds the methods provide for the estimated values, and their computational effort (since many are small systems). In this paper, we characterize, with a thorough parameterization, the possible scenarios where two-clock synchronization is to be solved, and then conduct a rigorous statistical study of both scenarios and methods. The study is based on exhaustive simulations run in a super-computer. Our aim is to provide a sound basis to select the best clock synchronization algorithm depending on the application requirements and characteristics, and also to deduce which ones of these characteristics are most relevant, in general, when solving the problem. For our comparisons we have considered several representative methods for clock synchronization according to a novel taxonomy that we also propose in the paper, and in particular, a few geometrical ones that have special desirable characteristics for the two-clock problem. We illustrate the method selection procedure with practical use-cases of sensory systems where two-clock synchronization is essential.

## 1. Introduction

In many sensorics applications, the data gathered by different devices must be fused in order to perform some task as a whole, e.g., localization [1], monitoring/surveillance [2], remote sensing [3], and many more (see for example surveys [4,5]). Time synchronization among these sensor devices, that are usually connected through non-deterministic media, is a fundamental requirement for this purpose: data from different sources should share a common notion of time, i.e., a unique and consistent reference clock, in order to be correctly merged.

There are numerous clock synchronization methods devised for general topologies of transmissions: they can be found almost half a century ago in the case of network computing [6], and decades ago in the case of wireless sensors [7,8]. All of them consist of exchanging messages among the devices until the estimates converge. The final accuracy of these estimates is subjected to the inherent uncertainty in communications [9,10,11]; better average accuracies can be achieved if probabilistic methods are used, but at the expense of not having hard guarantees for the worst case [12].

In the core of time synchronization methods there is still the problem of synchronizing two clocks [5]. Solving this problem involves exchanging messages between the devices in order to reach a shared meaning of time (This is internal synchronization [13], as opposed to external synchronization, where the clocks are synchronized w.r.t. some time external to the system.). Those exchanged messages should contain non-decreasing monotonic timestamps of the corresponding local clocks, to be processed in order to estimate their relation.

**Figure 1:**
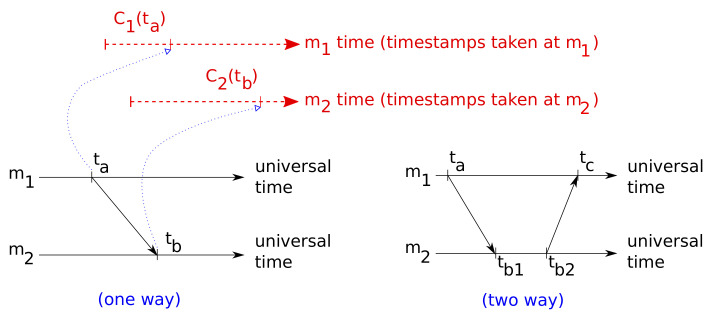
The most common message exchange schemes for clock synchronization: (**left**) one-way, (**right**) two-way. Note that the local time of each device (**top**) can start at any point in the universal time line and tick at any rate.

There exist several schemes for messaging, e.g., two-way message exchange, one-way message dissemination, or receiver-receiver synchronization [14]. Methods based on one-way messages only (Figure 1-bottom-left) are usually designed to take advantage of broadcasting [15,16,17,18], which is only available in general connected networks—sometimes broadcasting has to be implemented at the link level [12], something that requires significant support from the hardware as well. It is known that with a pure, non-reciprocal one-way message exchange scheme, it is impossible to estimate the clock offset and fixed delay precisely [19]. Therefore, the most relevant and meaningful scheme for two-clock synchronization becomes two-way exchange (Figure 1-bottom-right), that dates back at least to three decades ago [11]. Notice that in a sensory application it is not complicated to piggy-back sensory messages with the required timestamps, obtaining such a communication scheme without much modification to existing software; this fits particularly well in the case where a central device gathers sensory data from the rest periodically.

In the two-way scheme, one device ($m_{1}$) sends a message to the other ($m_{2}$) and waits for a response; both record and annotate in their messages the timestamps corresponding to the arrival and departure of measurements according to their local clocks: $C_{1}\left(t_{a}\right)$, $C_{2}\left(t_{b1}\right)$, $C_{2}\left(t_{b2}\right)$, and $C_{1}\left(t_{c}\right)$. After the exchange, $m_{1}$, that has gathered the four timestamps, uses them for improving its current estimation of the relation between both clocks.

That relation, although appearing in the literature under several forms and names, can be formalized with two quantities: the relative drift, that we denote as $\alpha_{R}$, which relates the deviations of the frequencies of both clocks from their nominal values, and the relative offset, $\beta_{R}$, that reflects the difference between their starting times—their zero-time timestamps—conveniently adjusted by their drifts. Formally, the two-clock synchronization problem can be stated as,

(1) $$\forall t,\;C_{2}\left(t\right)=\alpha_{R}C_{1}\left(t\right)+\beta_{R}+\varphi_{R}\left(t\right),$$

where it can be shown that [20],

(2) $$\begin{array}{cc} \alpha_{R} & =\left(r_{2}\tau_{1}\right)/\left(r_{1}\tau_{2}\right) \end{array}$$

(3) $$\begin{array}{cc} \beta_{R} & =\left(r_{2}/\tau_{2}\right)\left(t_{0,1}-t_{0,2}\right) \end{array}$$

(4) $$\begin{array}{cc} \varphi_{R}\left(t\right) & \in (-r_{2},\left(r_{2}\tau_{1}\right)/\tau_{2} \end{array}$$

being the following the basic parameters of the problem formulation:

- $r_{1}>0$, $r_{2}>0$ are the nominal clock periods of both devices;
- $\tau_{1}>0$, $\tau_{2}>0$ are the actual clock periods;
- $t_{0,1}\in R$, $t_{0,2}\in R$ are the universal time moments when both devices started their respective clocks;
- $\varphi_{R}\left(t\right)$ is the error produced by using discrete timestamps.

This formalization is valid as long as we assume that during the typically short time that passes between consecutive steps of any synchronization algorithm, i.e., between periodic requests from sensory data, the local clock in charge of timestamping at the *i*-th device is a linear function of the “universal time” t∈R, i.e., an affine transformation $\Phi_{i}\left(t\right)=\frac{t-t_{0,i}}{\tau_{i}}$. The result of this function can be discretized to model the timestamp (also inducing $\varphi_{R}\left(t\right)$) in this way: $C_{i}\left(t\right)=r_{i}\left\lfloor \Phi \left(t\right)\right\rfloor ,\forall t>t_{0,i}$.

Sufficient stability of $\tau_{i}$ in short periods of time has been recognized by several studies [21] (Typical crystal oscillators are accurate on the order of one part in 104 to 106 [12], although some authors have reported wider ranges, from −750 ppm to 750 ppm [21]. These frequency accuracies are known to be related to the possible bounds of the clock skew.), although there are algorithms that have explored more sophisticated models [18,22] and coped in a natural way with the variation of frequency (named skew) through signal processing [14]. However, these approaches do not provide hard guarantees due to their statistical nature. Note that, if required, variations in $\tau_{i}$ can be treated in deterministic methods without changing their fundamental design, for instance through moving windows on the sequence of two-way messages [12,23].

Far from being a simplistic view if considering generally connected systems, the two-clock synchronization problem explained so far, which is the focus of this paper, is of paramount importance in applications whose transmissions do not form arbitrary topologies but consist of a number of sensor devices transmitting data only in pairs—often wired—for instance, the sensory apparatus of a mobile robot, the set of sensors in a factory cell, remote surveillance applications, or home automation.

The primary interest when performing pairwise clock synchronization, especially for small and critical applications, lies in:

1. The quality of the estimation: accuracy and precision.
2. The existence of hard guarantees, i.e., strict bounds, in the resulting estimates, particularly important for critical systems.
3. The applicability of the methods to the special needs of these systems: limited computational cost, the shape of the transmission delays distributions, etc.
4. The number of estimated parameters that relate both clocks.

Providing hard guarantees on the estimates (point 2) and, simultaneously, a reasonable accuracy and precision in the results (point 1) is a complex matter, especially if we are also interested in the topics highlighted in point 3 above, as it happens in many pairwise applications. In this work, we aim to analyze all of these aspects from a practical perspective.

Due to the large number and diversity of existing clock synchronization methods, the first contribution of this paper is a concise taxonomy focused on the points of interest listed above, i.e., aimed at sensory applications where two-clock synchronization is sufficient. We have placed diversity of existing algorithms into that taxonomy, and, based on that, selected a number of representative ones for the subsequent study.

Due both to the diversity of algorithms and the difficulty of deducing analytically, in closed-form, their performance (some methods do offer analytical results for their bounds, but most do not have any analytical form for their average results, for instance), the second contribution of this paper is a statistical, yet rigorous, analysis of both methods and scenarios. We characterize pairwise synchronization scenarios with a number of parameters chosen to reflect a diversity of applications, ranging from clock frequencies to transmissions delays. For gathering data for the statistical study, we have carried out exhaustive simulations in a super-computer —they would have been difficult to run in a reasonable time and with the same thoroughness in conventional machines—, from which we have drawn a number of conclusions.

Our study of the simulation results includes a statistical comparison, based on ANOVA [24], of the performance of the selected methods in that variety of scenarios. We use a number of measures of performance for our comparisons, strongly related to the previously-listed aspects of the quality of the estimates, computational cost, etc. This statistical analysis has identified which method performs better in each aspect, but also which parameters of the scenario, i.e., the particular sensory system, have the strongest influence on these measures.

Finally, and using these results, we also contribute with some guidelines to select the best synchronization method for each situation, and illustrate them with a number of practical sensory applications where two-clock synchronization appears.

The main contributions of this paper are, in summary:

- A taxonomy of methods defined from the perspective of the essential aspects of two-clock synchronization applications listed above.
- A highly parameterized and detailed characterization of scenarios where two-clock synchronization can be carried out.
- A rigorous, thorough statistical comparison of a number of representative two-clock synchronization methods run in simulated environments synthetically generated from the previous characterization and evaluated with a set of performance measures that cover their applicability, quality of estimation and existence of hard guarantees.
- A study of which characteristics in the sensor scenario are statistically relevant for the two-clock synchronization problem and guidelines to select the best synchronization method for a given distributed sensor system, illustrated with some practical use cases.

The structure of the rest of the paper is as follows. Section 2 gives an overview of the two-clock synchronization problem, introduces a concise taxonomy of methods applicable to synchronize pairs of clocks—even when many were devised originally for general networks—that is focused on the most relevant aspects of pairwise systems, and justifies the choice of the representative algorithms used in further comparisons. Section 3 describes the simulation framework, the statistical results obtained with it and their use in practical cases. Finally, we highlight the main conclusions of this work and outline future lines of research.

## 2. Related Work and Synchronization Methods

In this section we propose a taxonomy of existing synchronization methods from the perspective of two-clock synchronization, also describing briefly those selected for the analysis of later sections.

In order to classify the existing methods that can be used for synchronizing two clocks according to the most relevant aspects for pairwise systems, explained in the introduction, we propose, in the following, a concise taxonomy (regarding the wide diversity of existing methods) formed by distinguishing whether the method:

1. produces some measure of uncertainty along with the estimates. Many methods do not produce any uncertainty measure and therefore the degree of belief in the estimate cannot be completely assessed [6,11,12,15,18,21,23,25,26]. In the case of those that use consensus in a network, some measure of their convergence to a common clock may exist, but that does not indicate the precision of such a clock either [4,16,17].
2. provides hard guarantees on the estimates versus soft guarantees or none at all. The former are so-called deterministic; they focus on the worst-case performance, some of them giving those guarantees for both $\alpha_{R}$ and $\beta_{R}$ [25,27,28], others only for $\beta_{R}$ [4,11,15,23] or indirectly [13,29]. Notice that hard guarantees lead to a measure of uncertainty in the estimates: the bounds can be interpreted as the extremes of a uniform distribution. On the other hand, methods that provide soft guarantees are so-called probabilistic and focus on the average performance [22,26], having usually better expected estimates than deterministic methods (e.g., microseconds vs. milliseconds errors [28]). Finally, among the methods that do not include in their design any guarantee on the accuracy or precision of their estimates are [12,16,17,18,19,21].
3. does not impose any particular constraints in transmission delays or in other aspects of the system. For instance, some methods assume bounded delays [4,11,15], particular forms for their probability distributions [19], or knowledge about their moments [26]. Assuming also that a bounded clock drifts [6,11,15,26,27,28] and/or offsets [6], or very short nominal clock periods w.r.t. network delays [26], is common and reasonable. Sometimes, an useful assumption is symmetric communications [11,13,23], i.e., the same delays in both directions of transmissions.
4. provides estimates for both $\alpha_{R}$ and $\beta_{R}$ versus only one. The methods providing both estimates can do it either directly [19,25,27,28] or indirectly [13,18]. The latter typically estimate $\beta_{R}$ [11,15,23], and only in rare occasions restrict to $\alpha_{R}$ [21]. Notice that, if the synchronization process is repeated frequently, estimating only $\beta_{R}$ can be enough; $\alpha_{R}$ becomes useful if synchronization is not so frequent, since it permits to predict the evolution of other devices clocks without communicating with them [27].

Items 1 and 2 are related to the quality of the estimates, while items 3 and 4 define the kind of situations where the synchronization can be used. To this concise taxonomy we could add the computational cost axis, but that can be roughly simplified by the fact that deterministic methods tend to be more computationally demanding than probabilistic ones, mainly because the latter are usually devised for networks with not very powerful computing devices. Notice, though, that the availability of hard guarantees, only available in the former, is relevant for many pairwise systems.

Since comparing and analyzing the performance of every method referenced above is out of our possibilities, we have selected for the study in this paper a reduced number of algorithms that cover well the variety defined by the proposed taxonomy. We briefly comment on them in the following.

The well known NTP [23] is relevant for its pervasivity in computers connected to the Internet, for its low-cost implementation, and for representing many approaches designed in the mid-nineties for wired networks. It consists of diverse modules, among which there is a very efficient algorithm to estimate the relative offset of two clocks, i.e., $\beta_{R}$. NTP does not estimate $\alpha_{R}$, but disciplines clocks for achieving $\alpha_{R}≃1$. The offset estimation algorithm works with a moving window for better adapting to changing drifts. It does not provide any measure of uncertainty in the estimate, but it usually achieves millisecond precision [22]. Its estimations assume symmetric communications. An evolution of NTP is PTP (“Precision Clock Synchronization Protocol”, IEEE standard 1588 [30]); although this protocol can provide much better synchronization than NTP (below microseconds [22]) under the same symmetry assumption, it is used in special-purpose industrial automation and measurement networks since it requires specific hardware (e.g., high-speed Ethernet LAN) with automatic timestamping, which is prohibitive for most pairwise systems.

Berthaud’s algorithm [27] is an excellent representative of the so-called “geometrical” approaches [19,25,27,28], built upon earlier frameworks (The use of convex polygons and estimation of clock functions as tangents to them was first proposed by [31], according to [28].) like [32], who exploit an intuitive spatial interpretation of the mathematical synchronization problem. Such a geometrical perspective is attractive because a linear bi-parametrical setting based on unknowns $\alpha_{R}$ and $\beta_{R}$, along with linear constraints existing between time measurements gathered in successive message exchanges, can serve to define polygons in a mathematical plane that provide the hard guarantees: the true values of the unknown parameters, i.e., a point, must lie within them. That point varies as successive messages shrink the polygons, ensuring convergence to the final estimate. Berthaud’s algorithm is “indirect” since it defines the mentioned polygons in the plane of measured local times instead of the $\alpha_{R}$/$\beta_{R}$ plane. An issue with this is that once a suitable bounding region is defined on the former, the estimates for the parameters $\alpha_{R}$ and $\beta_{R}$ must be deduced through additional mathematical transformations and therefore computational cost. Another one is that even when hard bounds are provided for the estimates, they can usually only be transformed into rectangular intervals in the $\alpha_{R}$/$\beta_{R}$ plane, ending in very conservative guarantees (larger uncertainties). Moreover, Berthaud’s method assumes a maximum value for the relative drift and of constant drifts during estimation—the latter is easily relaxed if the method uses a moving window of messages. Its computational cost is generally high.

The geometrical algorithm presented in [20], that can be abbreviated as DGP for Direct Geometrical Pairwise synchronization, shows similar features in the estimation of $\alpha_{R}$ and $\beta_{R}$ as that of Berthaud’s, but it runs a more computationally efficient procedure due to its direct work on the $\alpha_{R}$/$\beta_{R}$ plane. One of the first “direct” geometrical approaches was [19], that reported a nice statistical solution in the form of maximum likelihood estimator, however without hard guarantees and under the assumption of a very particular form for the probability distribution of the communication delays (exponential), specifically aimed at wireless sensor networks. In general, communication delays, that are the composition of diverse terms [12], are likely to be produced by heavy-(right)tailed distributions such as the lognormal [33,34] or the log–logistic [35]. Thus, a less restricted approach is needed which is applicable to pairwise systems. The DGP method that we include in this study does not require any particular form for the delays distribution and includes hard guarantees along with the estimates [20].

We also include in our comparisons a variant of DGP called DGP-α1: it assumes, as an additional restriction, that $\alpha_{R}=1$, i.e., that both clocks have equal drifts, and thus it only estimates $\beta_{R}$. This can be a reasonable assumption in systems where the pairs of devices are placed within a relatively small physical area (embedded systems) and thus share similar environmental conditions, and/or when they are mounted with similar quality components. Systems that use some clock disciplining (whose result will be the regulation of $\alpha_{R}$ around a desired value of 1), for instance, those that include NTP, can be a proper base for this variant. In all those situations, the results of this method are even more computationally efficient than DGP while providing comparable quality—achieving a performance that is quite close to the fastest, non-polygonal synchronization algorithms—while maintaining the quality of the estimates and the hard guarantees of geometrical approaches.

Finally, linear regression is a very simple and efficient approach to estimate the relation between two clocks, and it has been used by several time synchronization methods (e.g., FTSP [36]), especially in wireless sensor networks, where the computational power is limited [12]. Due to its nature, it cannot provide any guarantees or measures of uncertainty in the estimates, though it can serve for estimating both $\alpha_{R}$ and $\beta_{R}$ and it does not impose special constraints on the system (again, it can be adapted to working with a moving window of messages to increase its robustness against changes in drifts). Among its disadvantages, it is recognized in the literature that regression alone is not enough for performing a good estimation [21]. We include it here as a simplistic reference for several measures of performance.

## 3. Statistical Analysis of Methods and Systems

This section is structured as follows. In Section 3.1 we describe both the simulation and the statistical frameworks devised for analyzing methods and systems in the pairwise synchronization problem statistically. Section 3.2 is devoted to the study of how the pairwise system characteristics influence the synchronization results, particularly the average performance of synchronization, and Section 3.3 to the comparison of the methods. Finally, in Section 3.4 we provide guidelines based on these results that serve to choose the best clock synchronization method for a given application, and illustrate that selection with practical examples.

### 3.1. Overview of the Simulation and Statistical Frameworks

We have designed a simulation framework both to have a reference true value of ($\alpha_{R}$, $\beta_{R}$) with respect to which we can measure the quality of the estimates, and to easily set up a wide diversity of pairwise sensor systems (scenarios) where the methods described in Section 2 are run a large number of times for their performance to be statistically compared.

Our framework is based on a thorough parameterization of scenarios: it allows us to set a number of configuration axes at different independent positions in order to define the particular scenario to test (see Table 1). The first five axes configure the true clock relation existing between the two devices, $m_{1}$ and $m_{2}$, i.e., they completely set $\alpha_{R}$ and $\beta_{R}$, while the rest configure the transmission and execution characteristics of the pairwise application. For simulating round-trip times realistically we use a three-parametric log–logistic distribution, that has shown better modeling capabilities of actual round-trip times than other marginal probabilistic models in a variety of real networked systems [35,36,37]—it is more flexible than the log-normal, and can approach heavy-tailed, exponential-like and even Gaussian-like distributions very closely.

We can independently define any continuous position in each axis within two given extremes. We have chosen for our experiments extreme values that cover a wide variety of pairwise systems (last column of Table 1). For instance, most microcontrollers for embedded systems in the market use clock frequencies in the range of tenths-hundreds of MHz, thus we have set extreme values in the axes to explore scenarios that have clocks ranging from 10 MHz to 500 MHz. Since clock drifts can vary a lot—although they are normally kept in low values—we have considered nominal period deviations from those clocks ($\alpha_{i}$) going from a half to double. Starting times of clocks can occur very diversely; we have considered an accordingly wide range of offsets ($\beta_{R}$): within ±10 min. The delays inserted between consecutive two-way message exchanges, something that the programmer may choose in wide ranges too, have been set from 1 ms to 0.1 s. The proportions defined by axes $PE_{net/m_{2}}$ and $PV_{net/m_{2}}$ have been set slightly away from both 0 and 1 just to avoid degenerate cases of instantaneous times, but otherwise, they cover all the possibilities. Notice that the Symmetry axis goes from 0.5 to 1 instead of 0 to 1 because for the formal specification of the clock synchronization problem the asymmetry existing between communications in the $m_{1}\to m_{2}$ and $m_{2}\to m_{1}$ directions is not distinguishable, i.e., it does not matter where the longer delay occurs; again, the chosen range covers all the possible situations.

We also impose two additional, minor constraints on the scenario defined by the position of the configuration axes: $r_{i}$ must be multiple of 1 ns (we work with timestamps that have 32 bits seconds and nanoseconds parts, which is common in general-purpose operating systems), and the minimum one-way time and the minimum execution time in $m_{2}$ are both lower-bounded by 1 μs (Although modern computers reach nanoseconds in effective—average—computation time and define data structures with such resolution for storing timestamps, that is not achieved reliably due to the high dispersion caused by inner CPU modules such as caches, branch predictors, reorder buffers, and because of the bottlenecks of I/O hardware and OS software.).

Once the configuration axes are assigned concrete values, i.e., a particular scenario is completely defined, the framework can simulate a number of two-way message exchanges between $m_{1}$ and $m_{2}$ (the first $t_{a}$ is taken as $max(t_{0,1},t_{0,2})+Gap$), launch the synchronization algorithms on these exchange messages, and calculate a number of performance measures. A sequence of two-way message exchanges in a given scenario is there alled an experiment. We ran experiments of 1000 two-way exchange messages, which was long enough for all algorithms to settle their estimates except for rare cases of divergence.

The performance measures we use on each experiment are defined in Table 2 according to the four points of interest listed in the introduction section: $M_{1}$ and $M_{2}$ are related to the speed of convergence of the algorithms; $M_{3}$ and $M_{4}$ give the quality of their estimations; $M_{5}$ through $M_{8}$ measure the computational cost in different forms; finally, $M_{9}$ through $M_{12}$ measure the amount of uncertainty associated to the estimates (some algorithms do not provide such information).

For $M_{1}$ and $M_{2}$ we use a procedure for detecting steady-state entry that is based on a smoothed derivative of the sequence of estimates: once that the derivative (slope) falls in absolute value below a certain threshold, i.e., the estimate becomes flat enough, and never grows above the threshold again, the algorithm is considered to be steady for that estimate. The rest of the measures are calculated with the last *S* iterations of the algorithms within the experiment, i.e., when it is reasonable to assume that it has reached steady-state; we have set S=10% of the total number of iterations within the experiment.

For the statistical analyses of both Section 3.2 and Section 3.3 we have simulated all the scenarios resulting from possible combinations of the two extreme positions of the axes of Table 1, doing a large number of experiments (2000) in each scenario. Then, we conducted an ANOVA on the collected measures (transformed by a log operation for improving the normality of the data [24] and analyzing each measure $M_{i}$ separately). Doing 2000 experiments per scenario allows us to obtain both high statistical power and a truthful model of the underlying probability distributions, and also to have enough data to form smaller subsets if ANOVA uses blocking, i.e., if it needs to split data. Since such a high power makes small differences in averages distinguishable for the ANOVA even when they are not actually that different, we also used ${\hat{\omega }}^{2}$ in our analysis, a measure of association strength, considering very relevant a strength ${\hat{\omega }}^{2}>0.1$—a threshold commonly used for representing medium/large strength in other contexts [38].

Although so many configuration axes give us great flexibility and fine control when simulating a diversity of scenarios, this becomes an issue regarding the time for running our exhaustive simulations. Even considering only the two extreme positions for each axis, as we have explained above, we got $2^{11}=2048$ possible combinations (scenarios); simulating one scenario out of the 2048, i.e., running 2000 experiments in it, having each experiment 1000 two-way message exchanges and launching the clock synchronization algorithms after each message exchange, takes around 1.2 h in a PC with an Xeon Intel processor @2.6GHz. This amounts to almost three and a half months of expected full-time computation for all the 2048 scenarios (We have implemented the code in Matlab and then generated self-contained executables with mcc. Coding directly in C or C++ would reduce the execution time by a small constant, which would not lead to a manageable total time either).

Our solution to this issue has been to work with the Picasso super-computer of the University of Málaga, that has completed all experiments for the 2048 scenarios in about 5 and a half hours, so we got a speed-up of almost 87x w.r.t. the sequential execution (This factor actually depends on the workload of the super-computer when the experiments are launched. These savings represent the case when all the experiments are run once, but we had to repeat the execution several times while debugging the code. All in all, we can estimate an approximate total reduction from more than a year of sequential work to less than one day). At the time these experiments were launched, Picasso delivered 74 TFLOPS and featured 4016 CPU cores (Intel E5-2670 processors at 2.60 GHz), 22.4 TB of RAM and 750 TB of disk. Its interconnection network is based on Infiniband QDR/FDR, and its work queue system, which allows us to submit several jobs at the same time, is based on Slurm [39]. Actually, we rely on Slurm array jobs in order to automatically enqueue all the 2048 scenarios with a single Slurm batch script, that contains:

#SBATCH --array=1-2048

The 2048 jobs are enqueued into one of the Picasso batch queues. Each job had access to its job_id via the SLURM_ARRAY_TASK_ID environment variable and therefore can simulate a specific scenario defined by that *id*.

### 3.2. Results (I): Influence of the Scenario on Clock Synchronization

We have first analyzed the data produced by the statistical simulations described in Section 3.1 to study which configuration axes of Table 1, i.e., which characteristics of the pairwise system scenario have a relevant influence on the measures of Table 2, unregarding the synchronization algorithm. Since some algorithms work with both $\alpha_{R}=1$ and $\alpha_{R}\neq 1$ but others only with the former, we perform separate analyses in the cases where the sets of applicable algorithms differ.

For this study, we have designed a between-within balanced ANOVA [24] for each configuration axis of Table 1, in which all experiments are divided into two blocks (the between factor) corresponding to each of the extreme values for that axis. Within each block, we merge the results of all the applicable algorithms. Our goal is to detect whether there are statistically significant differences in the average values of the measures $M_{i}$ of Table 2 when a fundamental characteristic of the application scenario—axis—changes from its minimum to its maximum.

The results are collected in Table 3, Table 4 and Table 5, where performance measures (first column) and configuration axes (gray shaded columns) are shown, except for those producing very insignificant results (${\hat{\omega }}^{2}<0.01$). Each entry $\left(M_{i},axis_{j}\right)$ shows, separated by ‘:’and rounded to the closest integer in its range whenever possible, the average value of the *i*-th measure obtained with the *j*-th axis at its minimum and at its maximum extremes respectively. We have highlighted in bold those that are above the association strength threshold ${\hat{\omega }}^{2}=0.1$, which are considered greatly relevant, while the ones with a modest relevance, i.e., ${\hat{\omega }}^{2}\in \left[0.01,0.1\right)$, have been left in regular face.

Our main findings can be explained from the content of those tables as follows:

- Estimating the relative drift of both clocks ($\alpha_{R}$) is faster (converges after fewer message exchanges) when round-trip times are short, i.e., when both communications and execution times in $m_{2}$ are short.
- Estimating the relative offset of the clocks ($\beta_{R}$) is faster if $\beta_{R}>0$, i.e., when $m_{1}$ starts its clock after $m_{2}$ (or, equivalently, if $t_{0,2}<t_{0,1}$), and also when round-trip times are short.
- The error estimating the relative drift ($\alpha_{R}$) is smaller in scenarios where the expected delay in communications is shorter than the expected execution time in $m_{2}$ (i.e., when $PE_{net/m_{2}}$ is small), and also when the gap between consecutive message exchanges is longer. Notice that the second condition, in principle, can be manipulated in any algorithm, since the gap is defined by the program in charge of message exchanges, although at the cost of needing a longer time to converge when it is increased.
- The error in estimating the relative offset ($\beta_{R}$) is smaller in three situations: when the round-trip times are shorter, when the communications delays are shorter than the execution times in $m_{2}$, and when the communications are symmetric in both $m_{1}\to m_{2}$ and $m_{2}\to m_{1}$ directions. Devices connected point-to-point with a high-speed full-duplex link, for instance, are likely to satisfy all of these.
- Differences detected in execution times of the algorithms when exposed to different scenarios are not highly significant, but in the case of geometrical methods, they are slightly more efficient in the number of polygon vertices in symmetric communication scenarios. Another result is that the number of vertices is kept around tenths, corroborating a hypothesis stated in [20].
- The total amount of uncertainty in geometrical algorithms, either measured as polygon areas or diagonals ($M_{9},area$ and $M_{10},diag$), and also the isolated amount of uncertainty in $\alpha_{R}$ and $\beta_{R}$ (which is not only produced by the geometrical algorithms), or, in other words, the inaccuracy of the algorithms, is smaller in scenarios with shorter round-trip times and with faster communications compared to the execution times in $m_{2}$.
- The clock drifts in $m_{1}$ and $m_{2}$, and the amount of uncertainty in the round-trip times, only produce modest effects in the synchronization problem: nominal periods in $m_{1}$ longer than the actual ones are of benefit for the convergence speed in the estimate of $\alpha_{R}$, for the error in that estimate, for the error in the estimate of $\beta_{R}$ in the case $\alpha_{R}\neq 1$, and for the amount of uncertainty in the estimation of both $\alpha_{R}$ and $\beta_{R}$ if $\alpha_{R}\neq 1$; on the contrary, nominal periods of $m_{2}$ shorter than the actual ones are good for obtaining faster convergence in the estimate of $\alpha_{R}$, for the error in that estimate, for the error in the estimate of $\beta_{R}$, and for the amount of uncertainty in both estimates; last but not least, deterministic scenarios, where variance in the round-trip times is smaller (including communications and execution times), produce smaller errors in the estimate of $\alpha_{R}$.
- None of the explored scenarios produce a detectable difference in the dispersion of execution times of the algorithms ($M_{8}$, std−comp), thus this is not shown in the tables. That jitter is not expected to be significantly influenced by the scenario, only by the CPU and OS executing the algorithms.
- Finally, $\tau_{1}$, $\tau_{2}$ (true periods of clocks) and $PV_{net/m2}$ (proportion of variance in transmission delays w.r.t. the one in execution times in $m_{2}$) have no detectable influence in the process of clock synchronization.

### 3.3. Results (II): Comparative Performance of Synchronization Methods

In this section, we show the results obtained in the simulated experiments described in Section 3.1 when comparing the methods for clock synchronization to each other. We are interested first in ordering the methods according to their average performance measures.

For that purpose, we have made a between-within balanced ANOVA design for each measure defined in Table 2. This time we have used the clock synchronization methods as treatments (within) and split the experiments data into blocks (between) aimed at optimising the unimodality of the data and therefore the reliability of the ANOVA. Doing this clustering, i.e., finding the blocks that exhibit optimal unimodality, involves two problems: potentially searching among all possible subsets of scenarios and calculating in each one some measure of unimodality.

Regarding the latter, we have used the Hartigan’s DIP statistic and its significance for the empirical pdf [40], for which public implementations exist [41]; the significance level of that test has been set to 0.001 to minimize the number of false-positive (apparent but not actual unimodal blocks).

The former subproblem (searching among all possible subsets of scenarios) is harder: the number of subsets of a given set is exponential in the cardinal of the latter, and, in this case, if we use as a basis for blocking the axes positions in the experiments, intractable ($2^{2048}$). For addressing that issue, we have implemented a heuristic search for optimizing the unimodality of the blocks that works like this: start with blocks produced by combinations of only the configuration axes that have some significant effect in the given performance measure (these axes have been identified in Section 3.2) and then perform hill-climbing by adding new varying axes, therefore increasing the number of blocks until no better unimodality is obtained for all of them. In practice, this method has worked very well except in a few cases where it stuck in local minima with poor unimodality; there we have mixed the automatic heuristic with an interactive, manually guided search.

The between-within designs formed in that way have passed two different statistical analyses to assess an ordering among the clock synchronization methods. The first one is a Tukey’s comparison [42], that, provided that an ANOVA test obtains strong enough results (high ${\hat{\omega }}^{2}$), deduces a statistically significant pairwise ordering. The second one consists of comparing each method results in each individual experiment to the results of the other methods, and tallying the proportion of times that the former is better (this has been called Common Language Effect Size (CLES) [24]). The CLES ordering can be interesting for particular applications, and it provides an alternative when the strength of the ANOVA results is not enough.

Notice that the number of possible orderings that can arise when comparing *n* synchronization methods to each other ranges from 2 (if n=2) to 120 (n=5), which is too much information that should be condensed to be properly understood. For simplifying those results, Table 6 shows only the best method in the ANOVA ordering, and Table 7 the best one in the CLES ordering. Algorithms are (gray shaded columns): DGP, Berthaud’s (B’s), DGP-α1, NTP, and regression (Reg). Each entry $\left(M_{i},alg_{j}\right)$ in the tables shows the percentage of blocks in the design where the *j*-th algorithm ranks the absolute best in the *i*-th measure. Notice that a method not ranking the best does not mean that it cannot be the best for particular scenarios, as we will see in more detail in the next section.

In Table 6 all the results except the $\alpha_{R}-entry$ measure for the B’s method have high significance with association strength greater than or equal to the 0.1 threshold. In both tables we use a light orange background for those algorithms that are the best in more than the 50% of the blocks; notice that the highlighted cells are mostly the same in both tables. Algorithms that cannot run or are not meaningful for a measure or $\alpha_{R}$ class have been left empty.

The main results of these tables can be explained as follows:

- In general, methods DGP and DGP-α1 ranked first in a large number of scenarios and measures. Particularly, they are the absolute best (the best in all scenarios, with strong statistical significance) concerning the amount of uncertainty in the estimates, regardless of how we measure that uncertainty. They are also the best considering the estimate error for the majority of scenarios. In the estimate error of $\beta_{R}$ when $\alpha_{R}=1$, all the algorithms find special difficulties to outperform the others.
- B’s method did not ranked first in any measure, although, as we will see in the next section, it is the best in particular kinds of scenarios. Concretely, DGP outranks it with strong statistical significance when measuring the complexity of the constructed polygons in all orderings and cases (scenarios), i.e., in the computational effort of the geometrical paradigm.
- NTP ranks first, with strong statistical significance and in a majority of scenarios, in the speed of convergence (# of message exchanges to reach steady state) in the estimate of $\beta_{R}$, a measure where, if $\alpha_{R}=1$, all algorithms find difficulties to outperform the rest. In those cases where it cannot be used (i.e., when $\alpha_{R}\neq 1$), it is the DGP method the one of choice. The smallest expected execution time ($M_{7}$, i.e., comp) corresponds to NTP with strong statistical significance in all scenarios, although when comparing each scenario individually (CLES) the best is the DGP-α1 variation. NTP always has the smallest jitter in execution times (std−comp).
- Regression only stands out in the speed of convergence to the estimate of $\alpha_{R}$.

### 3.4. Results (III): Guidelines for Selecting Methods

Table 6 and Table 7 provide a simplified view of the results concerning method comparisons. It would also be interesting to know, for particular scenarios (i.e., for practical applications of the two-clock synchronization problem), which method is best suited, and also to grasp some general idea of the magnitudes of the performance measures that the methods produce (The average performance values are only of secondary importance in this paper, though, since they come from the particular extreme values in configuration axes of Table 1, that have been selected for covering a wide variety of scenarios and not for reflecting what happens in any particular one).

Unfortunately, in our experiments we have 2048 different scenarios, because we have simulated all the possible combinations of extreme positions in the configuration axes of Table 1, and listing all the performance measures of each method in each scenario would be impractical; a more concise account of the results is required for reaching meaningful, generalizable conclusions. Therefore, instead of studying particular scenarios, we have partitioned all of them into clusters (in a reduced number, ranging from 5 to 8) for each performance measure $M_{i}$. Each cluster contains scenarios for which the synchronization methods produce a distinctive pattern in the magnitudes of $M_{i}$, what we have called a behaviour. After clustering, it is much easier and useful to draw meaningful conclusions for the behaviour observed in each cluster than considering individual scenarios.

In order to facilitate the identification of the scenarios contained in a given cluster, and thus to identify the pertinence of that behaviour for a given practical application where two-clock synchronization is to be solved, clusters have been labelled as described in the following. Each particular scenario in our experiments corresponds to a combination of extreme positions of the configuration axes, that can be either at their minimum values (‘−’ or ‘0’) or at their maximum (‘+’ or ‘1’), therefore it can be denoted as a unique binary number of 11 bits, one-bit position per configuration axis for an easier interpretation of the most important axes extreme positions. The reader can refer to Table 8 (where the columns labelled with ‘UC*’, meaning Use Case, will be explained in Section 3.4). A given cluster of scenarios can be denoted as a number using three digits: ‘+’ if all the scenarios in the cluster have that axis at the maximum value, ‘-’ if all have it at the minimum value, and ‘*’, if the axis has different values in different scenarios of the cluster. (Obtaining this three-digit number for a cluster of scenarios denoted as binary numbers can be done automatically if we cast it as the problem of finding the minimal simplification of a propositional logic formula. We have used for that purpose the exact Quine–McCluskey algorithm [43], particularly its efficient implementation in [44]). Such specification is simple enough for identifying the scenarios contained into the cluster concisely.

The partitioning of the 2048 scenarios into clusters for a given performance measure $M_{i}$ is hard, since the total number of possible clusterings in a set of 2048 elements is too large (Given by the Bell number [45]). Therefore, finding clusters that contain scenarios exhibiting a distinctive behaviour in the performance $M_{i}$ can only be done approximately. In our case, we have used an incremental procedure, starting by considering as a preliminary cluster each of the blocks of scenarios already found when optimizing unimodality in the analysis of the previous section; the rationale for this is that unimodality is clearly a sign of a compact, unique behaviour in a performance measure. Then, we have merged those preliminary clusters to each other as long as the result continue exhibiting, through visual inspection, similar behaviours in the magnitudes of the measure, until the smallest possible amount of clusters are left, reaching the number of 5–8 clusters commented before (Notice that merging blocks used in the results of Table 6 and Table 7 may hide orderings that were shown there. Concretely, this happens with the methods and measures collected in cells $\left(M_{2},DGP\right)$, $\left(M_{4},B^{\prime }s\right)$, $\left(M_{12},DGP\right)$ and $\left(M_{12},NTP\right)$).

**Figure 2:**
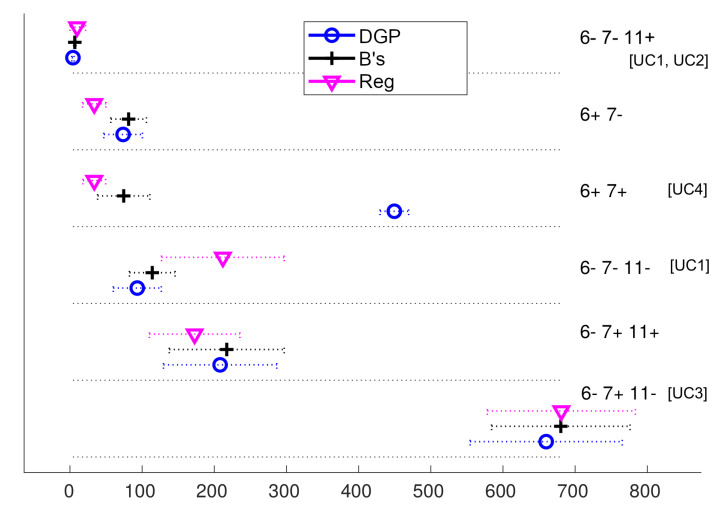
On the right side, from top to bottom, the six distinctive behaviours—clusters of scenarios—found for the $M_{1}$ ($\alpha_{R}-entry$) measure, arranged in six rows. The abscissa is the magnitude of that measure (number of iterations of the methods before entering steady-state). Each method within a behaviour has a point marker with its average value in the measure and ±1σ intervals around it (only illustrative of the dispersion, due to skewness). The lower the values, the better. The behaviours are labelled with the sets of scenarios that produce them (axes at their minimum extreme (−) or at their maximum (+) in all scenarios of the cluster). In square brackets, we also indicate the practical use cases that match the behaviours, from the ones described in Section 3.4.

**Figure A1:**
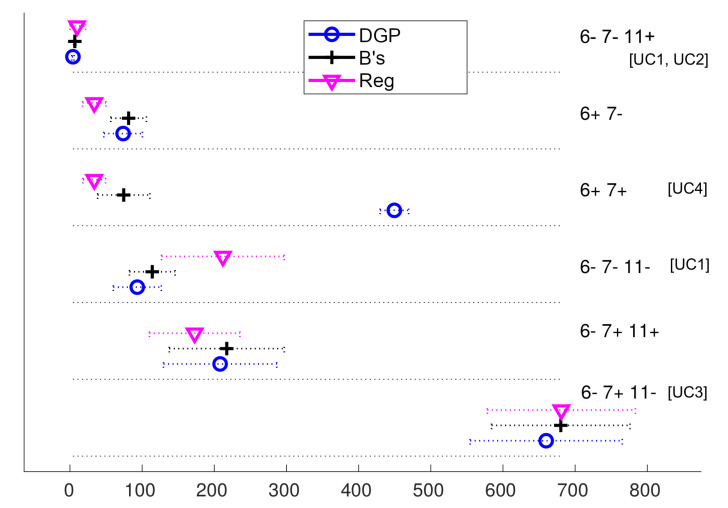
On the right side, from top to bottom, the six distinctive behaviours—clusters of scenarios—found for the $M_{1}$ ($\alpha_{R}-entry$) measure, arranged in six rows. The abscissa is the magnitude of that measure (number of iterations of the methods before entering steady-state). Each method within a behaviour has a point marker with its average value in the measure and ±1σ intervals around it (only illustrative of the dispersion, due to skewness). The lower values, the better. The behaviours are labelled with the sets of scenarios that produce them (axes at their minimum extreme (−) or at their maximum (+) in all scenarios of the cluster). In square brackets, we also indicate the practical use cases that match the behaviours, from the ones described in Section 3.4.

**Figure A2:**
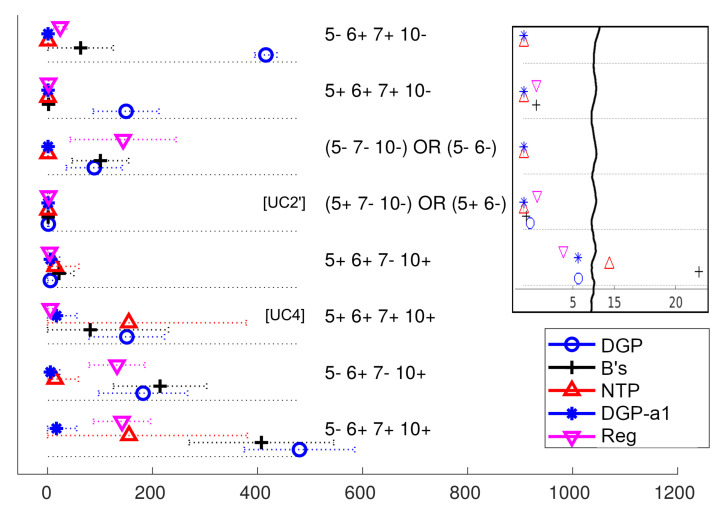
Eight distinctive behaviours have been found in the scenarios for $M_{2}$ ($\beta_{R}-entry$) when $\alpha_{R}=1$. Axes 3 and 4 must be at the same extreme position to assure $\alpha_{R}=1$. Top-right Zoom in on the average values at the left-most part of the first five behaviours.

**Figure A3:**
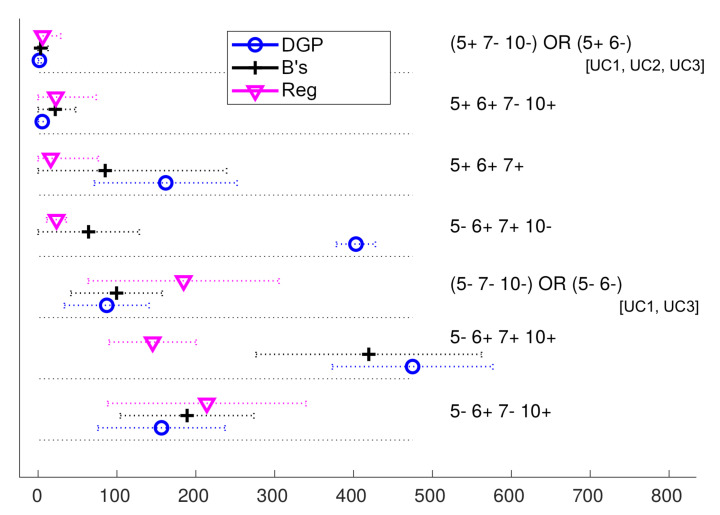
The seven distinctive behaviours found in the scenarios for $M_{2}$ ($\beta_{R}-entry$) when $\alpha_{R}\neq 1$ (consequently, axes 3 and 4 must be at different extreme positions).

**Figure A4:**
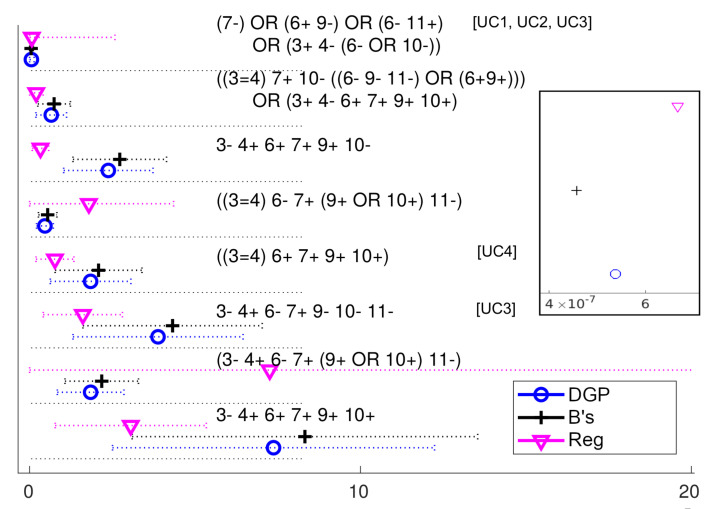
The eight distinctive behaviours found in the scenarios for $M_{3}$ ($\alpha_{R}-error$; its magnitude is in the abscissa). Right Zoom in on the first behaviour.

**Figure A5:**
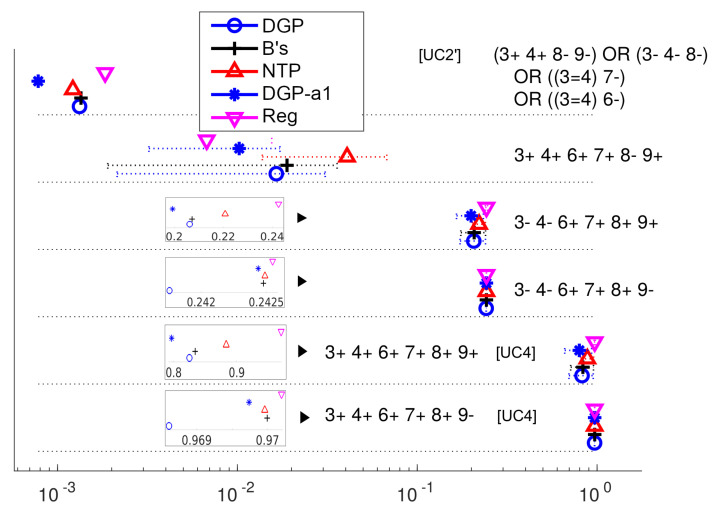
The six distinctive behaviours found in the scenarios for $M_{4}$ ($\beta_{R}-error$; its magnitude, in seconds, in the abscissa) when $\alpha_{R}=1$. The *x*-axis has been set to logarithmic scale for better observation of the small differences among algorithms in each behaviour. For the last four behaviours, additional zooms have been included.

**Figure A6:**
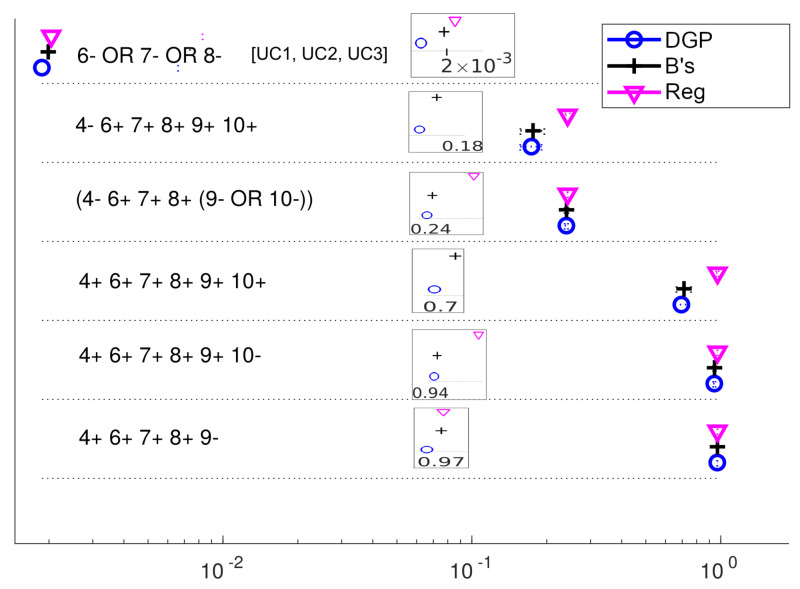
The six distinctive behaviours found for $M_{4}$ ($\beta_{R}-error$; its magnitude, in seconds, in the abscissa) when $\alpha_{R}\neq 1$ (axes 3 and 4 are at different extreme positions here). We have used a logarithmic *x* axis for better distinction between methods in each behaviour, and added some zoom.

**Figure A7:**
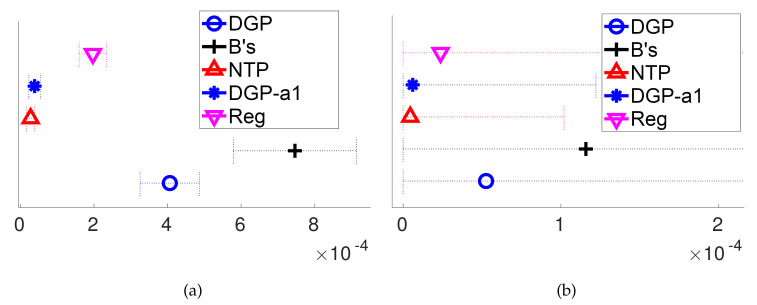
All scenarios produce a single behaviour for all methods both in (**a**) $M_{7}$ (comp) and (**b**) $M_{8}$ (std−comp). The magnitude of the measure is in the abscissa, in seconds.

**Figure A8:**
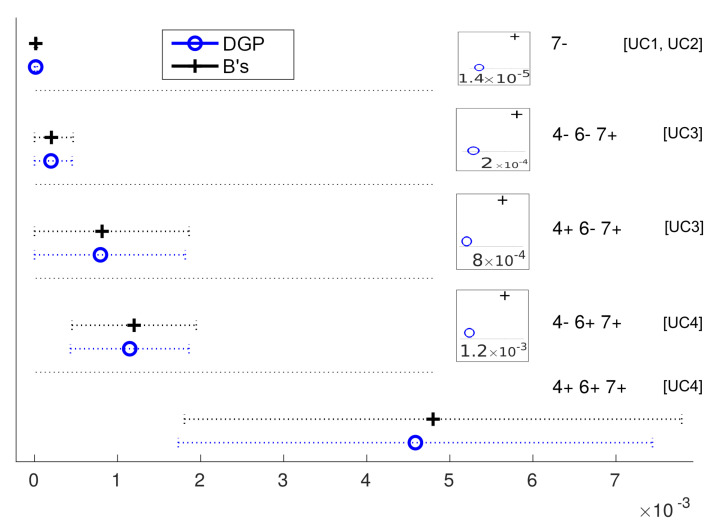
The five distinctive behaviours found in all scenarios for $M_{11}$ ($\alpha_{R}-uncert$), with zoom in those with more similar measure for both methods.

**Figure A9:**
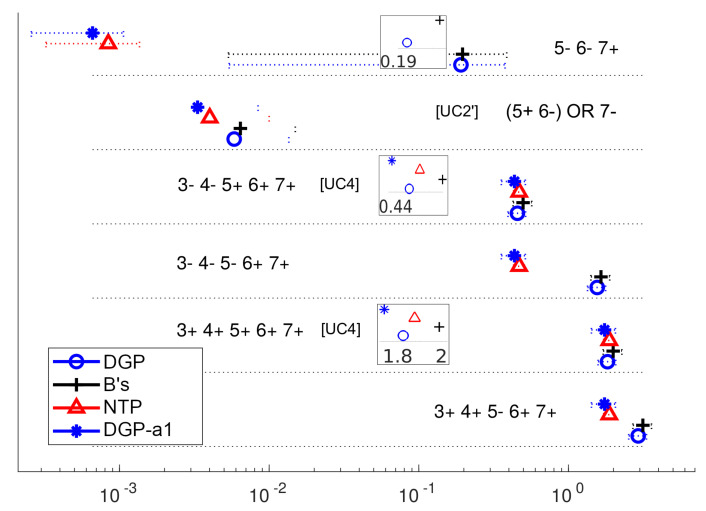
The six distinctive behaviours found in all scenarios for $M_{12}$ ($\beta_{R}-uncert$) when $\alpha_{R}=1$, with logarithmic *x* axis and zoom in some behaviours for better showing the methods differences. Axes 3 and 4 are always in the same extreme position.

**Figure A10:**
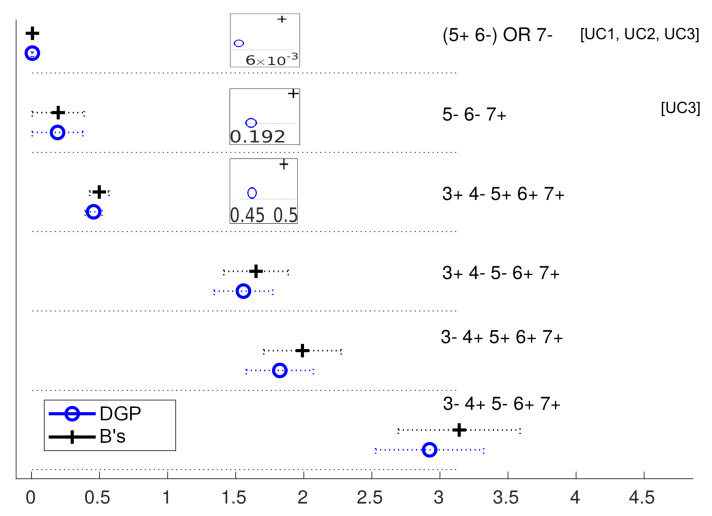
The six distinctive behaviours found in all scenarios for $M_{12}$ ($\beta_{R}-uncert$) when $\alpha_{R}\neq 1$ (axes 3 and 4 must therefore be at different extreme positions), with zoom in those with more similar measure for both methods.

Our procedure has produced the clusterings shown in the figures reported in Appendix A (Figure A1, Figure A2, Figure A3, Figure A4, Figure A5, Figure A6, Figure A7, Figure A8, Figure A9 and Figure A10): each figure corresponds to the clustering obtained on a given performance measure $M_{i}$, and shows the patterns that we have found, arranged in rows; each pattern is a given cluster or behaviour exhibited by all the synchronization methods in $M_{i}$. In Figure 2 we replicate the first of those Figure A1, corresponding to the $M_{1}$ ($\alpha_{R}-entry$) measure, for the reader’s convenience in order to illustrate their content.

These figures can be used as a guide to select the best two-clock synchronization algorithm for a given practical application in a quite direct manner: if the application configuration axes are at positions that match any of the behaviours shown in the figures for the performance measures of interest, the method of choice should be similar to the one with the best performance in those figures.

#### Use Cases

As practical examples of the use of Figure A1, Figure A2, Figure A3, Figure A4, Figure A5, Figure A6, Figure A7, Figure A8, Figure A9 and Figure A10 to select a synchronization method for a given application, we describe here a few common use cases (UC). The last columns of Table 8 summarizes them by indicating the position of the configuration axes that a scenario should have; in that table ‘≠’ abbreviates ‘3≠4’ (i.e., $\alpha_{r}\neq 1$) and ‘=’ abbreviates ‘3=4’ (i.e., $\alpha_{R}=1$); blank entries are not at any extreme in the corresponding UC; ‘*’ means that the extreme can be freely set at least in some particular instantiation of the UC.

Notice that the use cases we have considered here are very generic ones for the sole purpose of illustrating the utility of our statistical results (although their text can be translated directly to precise positions in the configuration axes of our framework); any particular application can vary in the indicated axes extremes. For completing the possibilities of use of our study in real cases, we finish this section with a particular use case in robotics.

- **[UC1] Embedded application**. This case of use represents a central embedded $m_{1}$ device (e.g., an SBC with a microprocessor) communicating with a number of slower microcontrollers that manage the rest of sensors (several $m_{2}$), all connected through a PCB short-length bus (e.g., I2C) and being powered up nearly simultaneously, with no provision for clock disciplining and not much computer power either. The gap between consecutive two-way message exchanges can be varied depending on the particular use of the system.
- **[UC2] Mobile robot**. A mobile robot is usually equipped with an on-board laptop with not-too-high computational power that gathers information from several sensors through USB connections (no network in between), being that all of these sensors are controlled by CPUs much slower than the one in the laptop. The sensoric hardware is usually powered up before the laptop is. Transmission times have a larger variance than in UC1 when we consider the non-real-time software in the laptop. The period of sensor/actuation sampling in a service mobile robot is usually of tenths of seconds, which limits the possibility of reducing the gap between consecutive two-way message exchanges beyond that. Since an important concern in mobile robots is the duration of their battery, computational savings are usually desirable.
- **[UC3] Domotic application**. Home automation hardware and software are particularly diverse since the market is still very fragmented [46]. Here we consider a very generic case where relatively fast and deterministic transmissions exist between the central hub/gateway, that we consider having a relatively high computational power, and the sensor devices, but those devices are faster in computing than transmitting. We also consider a short sampling period of the sensors and therefore a short gap between consecutive two-way message exchanges.
- **[UC4] Remote surveillance**. In remote surveillance a number of sensory devices are placed at a considerable distance from the central station, a modern computer capable of handling graphical interfaces, that samples them periodically. We assume an Ethernet connection that uses a general network to access the devices, i.e., routing with possible different paths in both directions, which is likely to produce long transmission delays with large variance and asymmetry. It is reasonable to consider the sensors to be powered up before the central station starts to work, and, since the transmissions are relatively slow, the gap between consecutive two-way message exchanges cannot be short. Unlike the previous use cases, all devices being a part of a network provides them with the possibility of disciplining their clocks, i.e., having $\alpha_{R}≃1$.

After consulting Figure A1, Figure A2, Figure A3, Figure A4, Figure A5, Figure A6, Figure A7, Figure A8, Figure A9 and Figure A10 looking for behaviours that match the UC configurations of axes shown in Table 8, the best method depending on which measure $M_{i}$ we consider more important is shown in Table 9. In that table we have indicated the recommended method on average and, in parentheses, which alternative should be selected, if available, that has a better average computational cost according to Figure A7. We have also added a variant of UC2 in that table (UC2’) that assumes some clock discipline is performed onboard the robot, i.e., $\alpha_{R}≃1$. In that case, both the DGP-α1 and the NTP methods produce the same $\beta_{R}-entry$ average results, with NTP having a slightly better average execution time.

**Figure 3:**
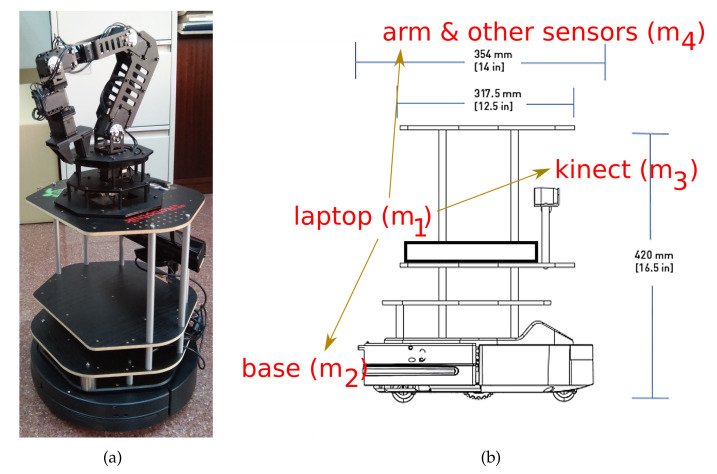
(**a**) CRUMB: a Turtlebot-2 robot composed of a Kobuki mobile base, a manipulator mounted on top and room for placing a laptop and other possible sensors [47]. (**b**) Star-like topology of the data transmissions in this robot, where the laptop acts as the central device.

As a more detailed example of the use of our statistical analyses for selecting a two-clock synchronization method, let us consider a particular case in robotics taken from the general paradigm of UC2: the CRUMB robot, a mobile system we employ in our research based on a Kobuki platform augmented with a manipulator and other sensors (Figure 3). This system exhibits the star-like transmission topology we have focused on in this paper, being that the laptop is a central device in charge of gathering data from multiple sensors and making decisions (navigation, mapping, pick-and-delivery tasks,…).

For illustrating the selection of a method for this system, we can consider the laptop ($m_{1}$) and the base ($m_{2}$), since the latter contains multiple sensory devices itself and the former can provide the data from the others. In that configuration, $m_{1}$ is an Intel Celeron @2.16GHz, which would yield a nominal clock resolution of 0.43 ns; however, since the OS (Linux) only provides nanosecond resolution, we take $r_{1}=1$ ns instead. That laptop is usually connected to the Internet and runs a clock disciplining software (embedded in the NTP protocol), thus we can assume $\alpha_{1}=r_{1}/\tau_{1}≃1\to \tau_{1}=1$ ns. On the other side, $m_{2}$ is an embedded computer that the manufacturer assures it does sensor sampling at a rate of 50 Hz; in particular, it is an STM32 256K high-performance microcontroller of a model undisclosed by the manufacturer but that corresponds to a clock of @72MHz in the information publicly available for that minimal specifications and family, which means that $r_{2}=14$ ns. Since this microcontroller has no clock disciplining, we can assume some drift for the clock resolution, e.g., $\alpha_{2}≃0.9\to \tau_{2}=16$ ns.

As for the transmissions, $m_{1}$ and $m_{2}$ are connected through USB 2.0, that provides 60 MB/s. The data transmitted in the $m_{2}\to m_{1}$ direction consists mainly of the information read from a bumper, a cliff detector, a wheel drop detector, a gyro sensor and the battery level; all in all, this amounts to about 20 bytes, that could be physically transmitted in 318 ns; adding a few bytes in the other direction and a minimal amount of $m_{1}$ software processing of the data, it would be typical an expected round trip time ($E_{rt}$) of a few hundreds of microseconds plus the time spent in $m_{2}$ for gathering the sensory data. Due to the processing in $m_{1}$, there would be some variance in the round trip time ($V_{rt}$; on the other hand, the microcontroller can be assumed to be practically deterministic); taking into account the tenths of millisecond resolution of task preemption in a standard Linux OS, we can consider that variance to be similar to the one of a uniform distribution for the [0,10] ms interval, i.e., $V_{rt}≃8\;\mu s^{2}$.

Transmissions between this pair of devices are quite symmetrical if we consider the few information going in any of the directions. The proportion of time spent in the transmissions plus the $m_{1}$ processing time must also be quite short compared to the time spent in the base microcontroller to gather the sensory data (1/50s=20 ms); that proportion ($P_{net/m_{2}}$) would then be around 0.01. Finally, the gap between consecutive exchange messages can be established as for any basic mobile robot operations, where it is typical to process sensory information every 100–200 ms, and we can also assume that the time between the base being started up ($t_{0.2}=0$ s) and the laptop finishing its loading of the OS ($t_{0.1}$) will be a few seconds, e.g., 10 s, which means that $\beta_{R}=0.9\cdot 10=-9$. Notice that if we would like to consider each of the sensors of the base individually (as a separate $m_{i}$), but using the same microcontroller, the only variation in all these specifications would be $t_{0.2}$ to represent the time offset between the sequential sensor data gathering.

In summary, our particular robotic system can be mapped into the positions of the axes shown in Table 10. The last two columns in Table 10 indicate, in a linear scale bounded to 0 to 1, the normalized position of the system in each axis if we take into consideration the bounds for the extremes given in Table 1, and the closest extreme to that position (‘-’ meaning ‘0’, ‘+’ meaning ’1’), respectively.

The data collected in the last column of Table 10 can now be directly used to select the best two-clock synchronization method for the system, by looking for those closest extremes in Figure A1, Figure A2, Figure A3, Figure A4, Figure A5, Figure A6, Figure A7, Figure A8, Figure A9 and Figure A10. As explained in the introduction, in a pairwise system we are interested in:

(i) The quality of the estimation: convergence (i.e., $M_{1-2}$), accuracy (i.e., $M_{3-4}$), and precision (mostly $M_{11-12}$).
(ii) The existence of hard guarantees: only DGP, DGP-α1 and B’s provide those.
(iii) The constraints existing in the system: computational cost, mainly $M_{7}$ and $M_{8}$.
(iv) The number of estimated parameters: both $\alpha_{R}$ and $\beta_{R}$, or only $\beta_{R}$ as it happens with NTP and DGP-α1.

We do not use here the rest of performance measures for their utility is related to more specific technical aspects, as explained in Appendix A.

In the particular case of this robotic system, we should use non-hard guaranteed methods only if they would provide better quality. The computational cost of the method will be supported by the laptop onboard the robot, thus it is important to reduce it as much as possible in order to reduce laptop battery consumption and thus to extend operational autonomy.

Table 11 shows the behaviours identified in Figure A1, Figure A2, Figure A3, Figure A4, Figure A5, Figure A6, Figure A7, Figure A8, Figure A9 and Figure A10 for the axes extremes listed in the last column of Table 10, and also which method performs better in each measure of interest. It is easy to see that the most distinctive measures are accuracy and computational cost; if we are concerned by computational cost—battery life—, and need a good quality in the estimate plus an estimation of both $\alpha_{R}$ and $beta_{R}$, the choice should be DGP. B’s would be the method of choice only if the accuracy of the estimation of $beta_{R}$ is crucial (at the cost of much more computational cost and slightly worse precision). Convergence to stable estimates is very similar for all methods in the case of $\alpha_{R}$ if the axis 5 is in its greatest extreme, i.e., if the mobile base is started up much longer before the laptop is, but clearly better in the case of DGP for more normal situations.

## 4. Conclusions

In this work, we have studied the problem of two-clock synchronization under the perspective of its application to sets of devices that communicate pairwise. There are relevant sensor applications where this form of synchronization is a core issue: embedded systems, robotics, domotics, etc. In such systems, clock synchronization is reduced to estimate the two main parameters that relate the clocks: $\alpha_{R}$ and $\beta_{R}$. It is of special importance to consider the uncertainty that the method has on the estimates (especially for embedded and critical systems), the rigidity of those estimates (hard vs. soft or stochastic), and its computational cost. Thus a particularized analysis of these systems is of special interest.

In the paper, we present a through statistical study that reveal some conclusions difficult or impossible to obtain from analytical approaches. We have selected, from the wide diversity that exists in the literature, a number of representative clock synchronization methods that can be applied to pairwise systems (some of them are commonly used as part of general network synchronization). Our selection of methods has been justified on a taxonomy that reflects the most relevant aspects of the problem in pairwise applications.

For carrying out a rigorous statistical analysis, we have implemented a rich simulation framework based on two-way message exchanges that allows us to represent most pairwise systems. It can be configured with a diversity of “axes”, simulates realistic transmissions between the devices, collects abundant data from the simulation in the form of performance measures, and conducts statistical tests, particularly ANOVA, to make comparisons.

Exhaustive simulated experiments have produced a number of interesting results that we claim can be useful both to detect which characteristic of the application are prone to impair/benefit any method, and which is the best synchronization approach for it.

Our analyses indicate that geometrical approaches produce the best results in many use-case applications of two-clock synchronization, and in important performance measures (being DGP-α1 preferrable if computational cost must be reduced). The well-known NTP method, provided that both clocks are disciplined, performs well if there are long transmission times with high uncertainty, as, for instance, in remote surveillance applications. More detailed results and implications have been commented in the previous sections.

In the future we plan to study the dynamics of pairwise clock synchronization, i.e., the case where the parameters to estimate vary over time, and also the effects of other dynamics of the system in the problem (e.g., abrupt changes of regimes in transmission delays). This will likely lead to new variants of the geometrical methods. Applications such as robotics have a special interest for us and therefore we will dive into their particularities and the role of clock synchronization in their architectures, a problem that it is often oversighted. 

## Figures and Tables

**Table 1:** Configuration axes for defining a pairwise scenario in simulation. $r_{i}$ is the nominal clock period and $\tau_{i}$ the actual one.

Axis	Name	Explanation	Extremes
1	$\tau_{1}$	True value of the period of $m_{1}$.	2:100 (ns)
2	$\tau_{2}$	True value of the period of $m_{2}$.	2:100 (ns)
3	$\alpha_{1}$	True value of $m_{1}$ clock drift, i.e., $r_{1}/\tau_{1}$.	0.5:2
4	$\alpha_{2}$	True value of $m_{2}$ clock drift, i.e., $r_{2}/\tau_{2}$.	0.5:2
5	$\beta_{R}$	True value of $\beta_{R}$ in seconds.	−600:600
6	$EM_{rt}$	Expected and minimum values for the round-trip time (total time in a two-way message exchange).	0.001:1 (s)
7	$PE_{net/m_{2}}$	Proportion of the expected and minimum transmission time within a round trip (the execution time in $m_{2}$ accounts for the rest).	0.01:0.99
8	Symmetry	Proportion of $m_{1}\to m_{2}$ expected and minimum times within the transmission time ($m_{2}\to m_{1}$ accounts for the rest).	0.5:1
9	$V_{rt}$	Variance in the round trip times.	4:40 (ms^2^)
10	$PV_{net/m_{2}}$	Proportion of the variance of transmissions w.r.t. the variance of $m_{2}$ execution times.	0.01:0.99
11	Gap	Delay between consecutive two-way message exchanges.	0.001:0.1 (s)

**Table 2:** Performance measures calculated in each experiment.

Label	Name	Explanation
$M_{1}$	$\alpha_{R}-entry$	Number of two-way message exchanges needed to reach steady-state in the estimation of $\alpha_{R}$.
$M_{2}$	$\beta_{R}-entry$	The same in the estimation of $\beta_{R}$.
$M_{3}$	$\alpha_{R}-error$	Average error in the estimation of $\alpha_{R}$.
$M_{4}$	$\beta_{R}-error$	Average error in the estimation of $\beta_{R}$.
$M_{5}$	vertex−mode	Mode of the number of vertices (only in polygonal algorithms).
$M_{6}$	vertex−max	Maximum number of vertices (idem).
$M_{7}$	comp	Average execution time of one iteration of the algorithm (secs).
$M_{8}$	std−comp	Standard deviation of the execution time of one iteration of the algorithm.
$M_{9}$	area	Average steady-state area of the polygons (average area of the rectangles formed by the hard guarantees in $\alpha_{R}$ and $\beta_{R}$ for the case of Berthaud’s).
$M_{10}$	diag	The same for the average diagonal length of polygons/rectangles.
$M_{11}$	$\alpha_{R}-uncert$	Average steady-state uncertainty in $\alpha_{R}$, i.e., distance between its bounds in hard guaranteed algorithms.
$M_{12}$	$\beta_{R}-uncert$	The same for $\beta_{R}$.

**Table 3:** Effects of the scenario on clock synchronization—clocks. Bold values are very relevant, as explained in the main text.

$M_{i}$	$\alpha_{R}$	$\alpha_{1}$	$\alpha_{2}$	$\beta_{R}$
$\alpha_{R}-entry$ (# msgs)		225:120	120:225	
$\beta_{R}-entry$ (# msgs)	1	32:39	32:39	**67:9**
	≠1			**135:21**
$\alpha_{R}-error$ (millionths)		6:2	2:6	
$\beta_{R}-error$ (thousandths)	1	29:116	29:116	73:72
	≠1	117:30	30:117	74:73
comp *(μs)*		240.4:240.3	242:239	
area (thousandths)		1.9:0.5	0.2:2.1	
diag (thousandths)			287:647	**633:301**
$\alpha_{R}-uncert$ (millionths)		1386:346	346:1386	
$\beta_{R}-uncert$ (thousandths)	1	183:531	183:531	423:290
	≠1	647:287	287:647	**633:301**

**Table 4:** Effects of the scenario on clock synchronization—delays.

$M_{i}$	$\alpha_{R}$	${\mathrm{EM}}_{\mathrm{rt}}$	${\mathrm{PE}}_{\mathrm{net}/m2}$	Symmetry
$\alpha_{R}-entry$ (# msgs)		**128:209**	171:241	
$\beta_{R}-entry$ (# msgs)	1	**18:55**		
	≠1	**36:111**		
$\alpha_{R}-error$ (millionths)		3.8:4.4	**0.4:7.8**	
$\beta_{R}-error$ (thousandths)	1	**1:143**	**1:142**	**2:141**
	≠1	**1:147**	**1:146**	**2:146**
vertex−mode (# vertices)		15:18	15:17	**14:18**
vertex−max (# vertices)		18:21	19:21	**17:22**
comp *(μs)*			238:243	232:249
area (thousandths)		**0.02:2.29**	**0.0001:2**	
diag (thousandths)		**49:884**	**8:926**	
$\alpha_{R}-uncert$ (millionths)		**254:1478**	**15:1717**	
$\beta_{R}-uncert$ (thousandths)	1	**20:693**	**6:708**	
	≠1	**49:884**	**8:926**	

**Table 5:** Effects of the scenario on clock synchronization—variances.

$M_{i}$	$\alpha_{R}$	$V_{\mathrm{rt}}$	Gap
$\alpha_{R}-entry$ (# msgs)			189:142
$\beta_{R}-entry$ (# msgs)	1		
	≠1		
$\alpha_{R}-error$ (millionths)		2:6	**6:2**
vertex−mode (# vertices)		17:15	
vertex−max (# vertices)		21:19	
area (thousandths)			1.2:1.1
diag (thousandths)			497:436
$\alpha_{R}-uncert$ (millionths)			1024:708
$\beta_{R}-uncert$ (thousandths)	1		
	≠1		497:436

**Table 6:** Orderings of the algorithms (ANOVA).

$M_{i}$	$\alpha_{R}$	DGP	B’s	DGP-α1	NTP	Reg
$\alpha_{R}-entry$		37.5	0			62.5
$\beta_{R}-entry$	1	18.8	0	12.5	62.5	6.3
	≠1	75	0			25
$\alpha_{R}-error$		55.5	0.8			43.8
$\beta_{R}-error$	1	6.3	2.3	79	0	12.5
	≠1	62.5	12.1			25.4
vertex−mode		100	0			
vertex−max		100	0			
comp		0	0	0	100	0
std−comp		0	0	0	100	0
area		100	0			
diag		100	0			
$\alpha_{R}-uncert$		100	0			
$\beta_{R}-uncert$	1	0	0	93.8	6.3	
	≠1	100	0			

**Table 7:** Orderings of the algorithms (CLES).

$M_{i}$	$\alpha_{R}$	DGP	B’s	DGP-α1	NTP	Reg
$\alpha_{R}-entry$		25	0			75
$\beta_{R}-entry$	1	65.6	0	12.5	21.9	0
	≠1	59.4	0			40.62
$\alpha_{R}-error$		55.5	0.8			43.8
$\beta_{R}-error$	1	6.3	10.9	68	0	14.8
	≠1	57.4	16.4			26.2
vertex−mode		100	0			
vertex−max		100	0			
comp		0	0	100	0	0
std−comp		0	0	0	100	0
area		100	0			
diag		100	0			
$\alpha_{R}-uncert$		100	0			
$\beta_{R}-uncert$	1	8.1	0	91.9	0	
	≠1	100	0			

**Table 8:** Interpretation of axes and use cases of method selection. ≠ stands for $\alpha_{R}\neq 1$, and = for $\alpha_{R}=1$.

Axis	Interpretation of the Axis Being ‘+’ (‘-’ Would Be the Opposite)	UC1	UC2	UC3	UC4
3 (4)	Clock freq. in $m_{1}$ ($m_{2}$) higher than nominal.	≠	≠	≠	=
5	$m_{2}$ clock started before $m_{1}$ clock.		+		+
6	Long round trips (transmissions + $m_{2}$ exec.).	-	-	-	+
7	Transmissions slower than $m_{2}$ exec.	-	-	+	+
8	High asimmetry in transmission times.	-	-	-	+
9	Large uncert. (variance) in round trips.	-	+	-	+
10	Uncert. in transmissions larger than in $m_{2}$ exec.		-	-	+
11	Long gaps between consecutive exchanges.	*	+	-	+

**Table 9:** Recommended synchronization methods for the use cases in Section 3.4.

UC	$\alpha_{R}$ Entry	$\beta_{R}$ Entry	$\alpha_{R}$ Error	$\beta_{R}$ Error	$\alpha_{R}$ Uncert.	$\beta_{R}$ Uncert.
1	D (R)	D (R)	B (D)	D (R)	D	D
2	D (R)	D (R)	B (D)	D (R)	D	D
2’	D (R)	D1(N)/N	B (D)	D1 (N)	D	D1 (N)
3	D (R)	D (R)	B (D) R (D)	D (R)	D	D
4	R	R (N)	R (D)	D (D1) D1 (N)	D	D1 (N)

*(D)—DGP; (D1)—DGP-α1; (B)—Berthaud’s; (N)—NTP; (R)—Regression*.

**Table 10:** Configuration axes positions of the CRUMB robot example.

Axis	Name	Value	Position [0…1]	Closest Extreme
1	$\tau_{1}$	1 ns	0	1-
2	$\tau_{2}$	16 ns	0.16	2-
3	$\alpha_{1}$	≃1	0.33	3-
4	$\alpha_{2}$	0.9	0.27	4-
5	$\beta_{R}$	−9 s	0.49	5−/+
6	$EM_{rt}$	20,200 μs	0.0192	6-
7	$PE_{net/m_{2}}$	0.01	0	7-
8	Symmetry	1	1	8+
9	$V_{rt}$	$8\;\mu s^{2}$	0	9-
10	$PV_{net/m_{2}}$	0.99	1	10+
11	Gap	200 ms	1	11+

**Table 11:** Selection of methods for the CRUMB robot example.

Measure	Name	Behaviour	Method Ordering
$M_{1}$	$\alpha_{R}-entry$	1st	$DGP<B^{\prime }s<Reg$, all similar
$M_{2}$	$\beta_{R}-entry$	1st if 5+, or 5th if 5-	$DGP<B^{\prime }s<Reg$, all similar if 5+
$M_{3}$	$\alpha_{R}-error$	1st	$B^{\prime }s<DGP<Reg$
$M_{4}$	$\beta_{R}-error$	1st	$DGP<B^{\prime }s<Reg$
$M_{7}$	comp	n/a	NTP<DGP-$\alpha 1<Reg<DGP<B^{\prime }s$
$M_{8}$	std−comp	n/a	NTP<DGP-$\alpha 1<Reg<DGP<B^{\prime }s$
$M_{11}$	$\alpha_{R}-uncert$	1st	$DGP<B^{\prime }s$, all similar
$M_{12}$	$\beta_{R}-uncert$	1st	$DGP<B^{\prime }s$, all similar

## Appendix A. Distinctive Performance Behaviours of the Synchronization Methods

This appendix lists all figures corresponding to the clusterings (=*behaviours*) obtained on the performance measures $M_{i}$ when comparing synchronization methods, showing the patterns that we have found in the methods arranged in rows. We do not include in those figures the clusterings for $M_{5}$ (vertex−mode), $M_{6}$ (vertex−max), $M_{9}$ (area) and $M_{9}$ (diag) because they only concern the comparison between the geometrical approaches (*DGP* vs. *Berthaud’s*), already covered in Table 3, Table 4 and Table 5, and in all observed behaviours the former method always beats the latter.

Please refer to Section 3.4 for the details of how these figures have been obtained and of their use for practical applications.
